# Mucosal IgA Antibodies are Critical for Bacterial Clearance of *Bordetella pertussis* in the Baboon Model

**DOI:** 10.20411/pai.v10i2.800

**Published:** 2025-06-13

**Authors:** Gaurav Chauhan, Melissa A. Gawron, Aaron J. Belli, Keith A. Reimann, Ryan Schneider, Yang Wang, Mark S. Klempner, Lisa A. Cavacini

**Affiliations:** 1 Department of Medicine, University of Massachusetts Chan Medical School, Worcester, Massachusetts

**Keywords:** *Bordetella pertussis*, Whooping Cough, Mucosal IgA

## Abstract

**Background:**

Despite the control of *Bordetella pertussis* with vaccine introduction, the incidence of pertussis has increased in the United States and globally. New vaccine strategies are clearly needed to regain control of this vaccine-preventable infection.

**Methods:**

Experimental pertussis infection of baboons induces an acute respiratory illness with clinical and laboratory features similar to whooping cough in man. In a previous study, acellular pertussis-vaccinated (aP) baboons were protected from clinical illness but not from prolonged airway colonization. In contrast, convalescent baboons are protected from both clinical illness and colonization. These studies suggest that current aP vaccines may be ineffective at preventing airway colonization, contributing to resurgence of pertussis.

**Results:**

In studies conducted at the University of Massachusetts Chan Medical School in Worcester, Massachusetts, mucosal IgG antibody responses in nasopharyngeal washes are similar in convalescent and vaccinated baboons. However, significantly higher mucosal anti-pertussis immunoglobulin A (IgA) responses are observed in convalescent animals.

**Conclusions:**

These studies suggest that mucosal IgA responses to some pertussis antigens will result in bacterial clearance.

## INTRODUCTION

The incidence of infection with *Bordetella pertussis*, the highly contagious etiologic agent for whooping cough, fell dramatically upon introduction of a whole-cell pertussis (wP) vaccine in the mid-20th century. While the wP vaccine nearly eliminated the public heath significance of whooping cough, injection site reactions, occasionally high fevers, and other more serious adverse events were seen. In the 1970s, concern over these side effects resulted in a global effort to develop an acellular pertussis (aP) vaccine that, specifically, lacked active bacterial endotoxin and pertussis toxin that were believed to be responsible for most of the unacceptable side effects. Acellular vaccines replaced whole cell vaccine in the US in 1997 based on demonstrations that they induced anti-pertussis immune responses and, more importantly, vaccine efficacy, with significant reductions in adverse effects. Unfortunately, the incidence of pertussis in the US and globally has gradually increased since the late 1990s, along with sporadic epidemics [1]. Accumulating studies have shown that titers wane more rapidly in aP vaccine recipients [2, 3] suggesting that failure of available vaccines to prevent infection may contribute to the reemergence of pertussis. Identification of pertactin-negative *Bordetella* in patients suggests that genetic variation in the organism, probably through selective pressure from vaccination [4–6], might also impact the resurgence of pertussis; however, that is not necessarily observed in countries where pertactin-negative Bordetella is circulating [7]. Overall, there is an acute need for a better understanding of the correlates of protective immunity against *B. pertussis*, leading to a more effective pertussis vaccine.

*B. pertussis* colonizes the murine respiratory tract; however, mice fail to recapitulate clinical whooping cough as seen in man. Development of a nonhuman primate model of pertussis using baboons (*Papio anubis*) has facilitated advances in understanding the pathogenesis and prophylaxis of pertussis. In this model, experimental inoculation of baboons with *B. pertussis* resulted in a highly reproducible replication of a whooping cough-like illness including fever, leukocytosis, and the characteristic, chronic cough [8]. Using this primate model [9], investigators have further demonstrated that immunization with aP prevented clinical disease upon challenge with *B. pertussis*. However, the respiratory tract of aP-immunized baboons remained colonized with pertussis organisms for up to 6 weeks after infectious challenge. Moreover, vaccinated but colonized baboons were able to transmit infection to naïve baboons through casual contact. In contrast, unvaccinated baboons that have recovered from experimental pertussis infection were protected from both clinical disease and airway colonization when rechallenged. Similarly, in a mouse model, immunization with the human aP vaccine prevents severe lung infections but does not significantly affect nasopharyngeal colonization, as *B. pertussis* can effectively colonize the mouse nasopharynx, spread within respiratory organs, evade robust host immunity, and persist for months [10]. Thus, studies in both a murine and baboon animal model of pertussis infection confirm that bacteria elimination cannot be induced through currently available aP vaccines. In the present study, mucosal antibody responses in vaccinated or convalescent baboons were analyzed. We demonstrate that mucosal IgA antibodies against pertussis antigens are key elements in the immune response seen in baboons with reduced or no colonization and should be considered as a goal for next-generation pertussis vaccines.

## METHODS

### Animal Studies

Nasopharyngeal washes (NPW) were provided by Dr. Tod Merkel and obtained from animals in the vaccine study described by Warfel et al [9]. Briefly, animals were inoculated intramuscularly with human doses of acellular vaccine (Daptacel, Sanofi Pasteur Ltd. or Infanrix, GlaxoSmithKline) for the aP arm (equal numbers of animals) and Triple Antigen (Serum Institute of India Ltd.) for the wP arm at 2, 4, and 6 months of age. The pertussis antigen content of each vaccine is listed in Table 1. Naïve animals were age-matched to vaccinated animals but did not receive vaccination. Convalescent animals were previously infected with *B. pertu*ssis but were clear of infection for at least 1 to 2 months prior to challenge.

**Table 1.** Pertussis Vaccines Used in Current Study

	DAPTACEL	INFANRIX	Whole Cell
Diphtheria toxoid	15 Lf	25 Lf	≤25 Lf
Tetanus toxoid	5 Lf	10 Lf	≥5 Lf
*B. pertussis*	-	-	≥4 IU
Inactivated Pertussis Toxin	10 µg	25 µg	-
Filamentous hemagglutinin	5 µg	25 µg	-
Pertactin	3 µg	8 µg	-
Fimbriae type 2&3	5 µg	-	-
Aluminum	0.33 mg	≤0.625 mg	≤1.25 mg

Daptacel is a product of Sanofi Pasteur; Infanrix is a product of GlaxoSmithKline; Whole Cell is a product of Serum Institute of India.

NPW were obtained as described previously [8] by flushing the back of the naris with 1 mL phosphate-buffered saline. The recovered washes from both nares were pooled and aliquoted. NPW was plated onto Regan-Lowe plates to determine the number of colony forming units (CFU), which were reported by Warfel [9]. NPW for antibody measurement were stored at -80 °C until assessed for reactivity with pertussis antigens by ELISA (see below).

Animal studies and procedures were conducted in a facility accredited by the Association for Assessment and Accreditation of Laboratory Animal Care International and in accordance with protocols approved by the Center for Biologics Evaluation and Research Animal Care and Use Committee. Principles outlined in the Guide for the Care and Use of Laboratory Animals by the Institute for Laboratory Animal Resources, National Research Council were followed.

### Measurement of Mucosal Antibody to *B. pertussis*

For antibody measurement, pertussis antigens were obtained from List Biological Laboratories and included fimbriae 2/3 (Fim, cat#186), pertactin (PRN, cat#187), pertussis toxin (PT, cat# 80), and adenylate cyclase toxin (ACT, cat#188). The measurement of baboon serum antibody has been previously described using some of these same antigens [11]. ELISA plates were coated with antigen at 0.5 µg/mL of carbonate/bicarbonate buffer pH 9.6 overnight and blocked using Superblock (ThermoFisher). Serial 2-fold dilutions of NPW were added to wells in duplicate or triplicate and incubated for 30 minutes. After washing, bound antibody was detected using goat anti-monkey IgG-HRP (Biorad, AbD Serotec AA142P) or monoclonal anti-baboon IgA-biotin (NIH Nonhuman Primate Reagent Resource, 9B9) (1 µg/mL) followed by Streptavidin-HRP. Antibodies were developed and/or confirmed to be specific for the Ig classes in *P. anubis*. After washing, TMB (3,3’,5,5’-tetramethylbenzidine, ThermoFisher) substrate was added and plates developed for 15 minutes prior to the addition of stop solution. Endpoint titers were determined as the last dilution of NPW with a signal greater than 2 times the negative control NPW. All data are reported as mean ± standard error of the mean.

### Statistical Analysis

Statistical analyses of ELISA data were performed by ANOVA with post hoc *t* test using GraphPad Prism. Box plot data includes the medians; box limits indicate the 25th and 75th percentiles as determined by R software; whiskers extend 1.5 times the interquartile range from the 25th and 75th percentiles; outliers are represented by dots; crosses represent sample means; and bars indicate 90% confidence intervals of the means.

## RESULTS

### ELISA Determination of Baboon IgG and IgA Antibodies to Multiple Pertussis Antigens

As noted in Table 1, several *B. pertussis* antigens are incorporated into the acellular vaccine. Both Daptacel and Infranix include inactivated pertussis toxin, filamentous hemagglutinin, and pertactin with the addition of fimbriae types 2 and 3 to Daptacel. While not quantitated, these antigens and several others are included in the whole cell vaccine (and upon natural infection). We selected 2 targets common to all vaccines and infection (PT and PRN), one that is present in some aP and in wP and infection (Fim) as well as one target involved in pathogenesis but only expressed in wP vaccine and natural infection (ACT). Based on published results using these antigens for the serological measurement of pertussis-specific baboon IgG [11], we used similar coating conditions and anti-Ig reagents confirmed to be specific for baboon Ig classes. Serum from a convalescent baboon was used to validate the ELISA for detection of baboon IgA (data not shown).

### Antibody Titers to Pertussis Antigens in NPW

**Figure 1.**
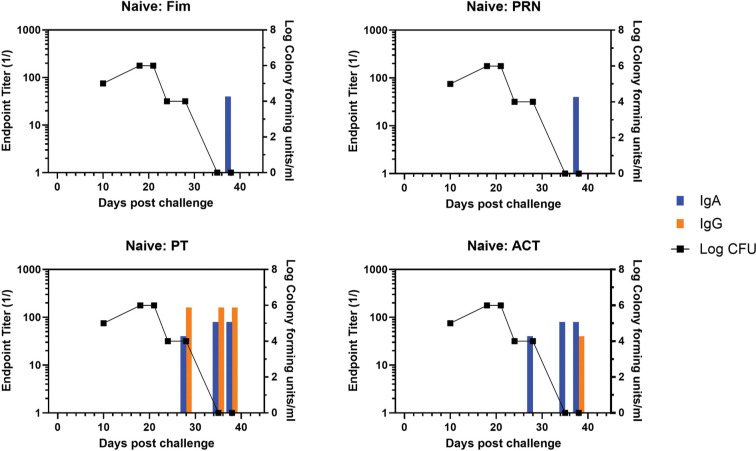
**Development of anti-pertussis antibody response and bacterial colonization in naïve baboons following challenge**. Endpoint titers of IgA (blue bar) and IgG (orange bar) to fimbriae (Fim), pertactin (PRN), pertussis toxin (PT), and adenylate cyclase toxin (ACT) in the NPW of a representative naïve baboon following challenge with pertussis is plotted against bacterial colonization (log_10_).

NPW samples were collected from all baboons utilized in the published nonhuman primate vaccine study [9] and included animal groups receiving aP (n=6) or wP (n=5) vaccine, convalescent (n=6) or naïve (n=5) at the time of challenge. IgG and IgA responses to pertussis antigens PRN, Fim, PT, and ACT were measured by ELISA using antibodies specific for either baboon IgG or IgA. The IgG and IgA endpoint titers against the 4 antigens were plotted against the CFU/mL of NPW. The temporal development of antibody responses (bar graphs) as well as clearance of infection (line graph) are shown for a representative naïve animal in Figure 1 with a heat map of individual responses in Supplementary Figure 1. Antibody to PT and ACT were measurable in NPW within 21 days whereas antibody to Fim and PRN were not detected until 31 days. Bacterial clearance was within 28 days.

At the time of challenge, aP-vaccinated animals had a good IgG response to Fim, PRN, and PT but no response to ACT, which is not an aP vaccine component.

**Figure 2.**
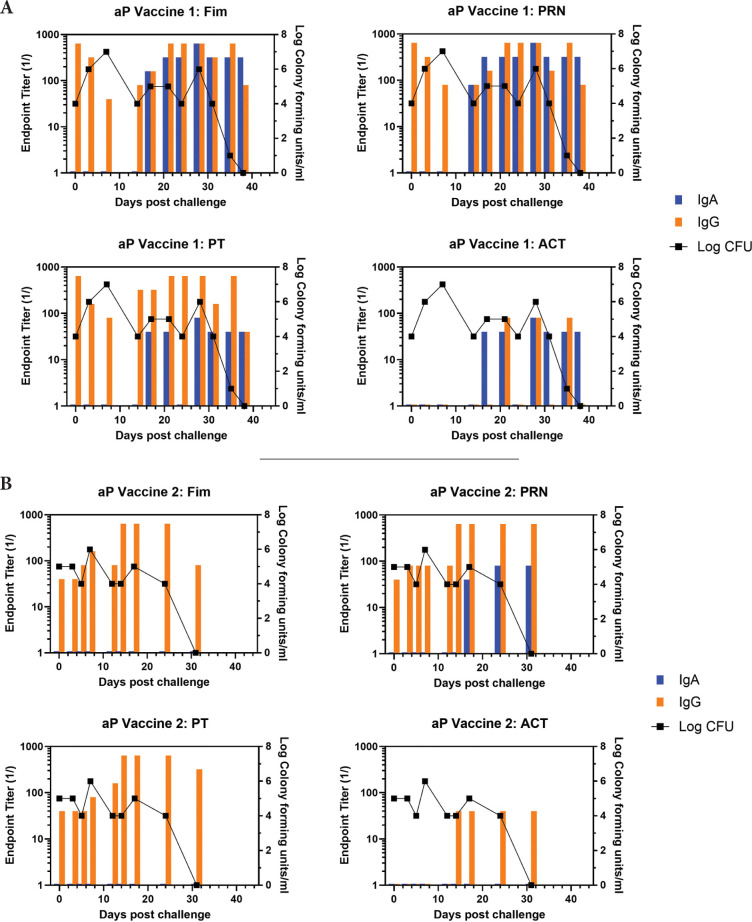
**Development of anti-pertussis antibody response and bacterial colonization in aP-vaccinated baboons following challenge**. Endpoint titers of IgA (blue bar) and IgG (orange bar) to fimbriae (Fim), pertactin (PRN), pertussis toxin (PT), and adenylate cyclase toxin (ACT) in the NPW of representative aP-vaccinated baboons (one from each commercial vaccine group labelled as aP Vaccine 1 and aP Vaccine 2) following challenge with pertussis is plotted against bacterial colonization (log_10_).

**Figure 3.**
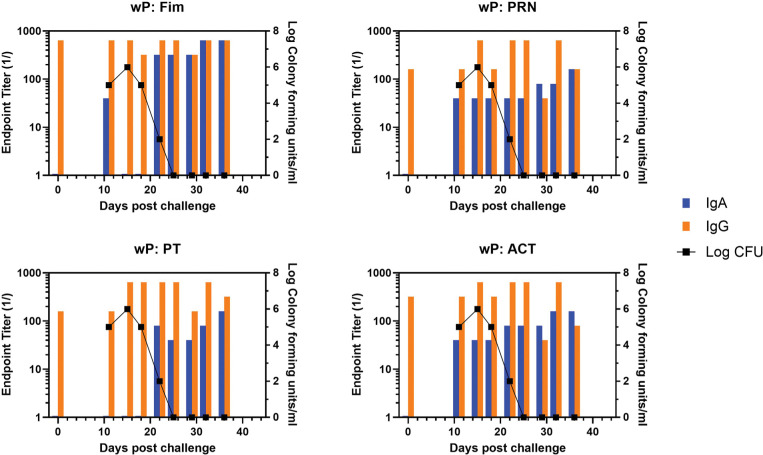
**Development of anti-pertussis antibody response and bacterial colonization in wP-vaccinated baboons following challenge**. Endpoint titers of IgA (blue bar) and IgG (orange bar) to fimbriae (Fim), pertactin (PRN), pertussis toxin (PT), and adenylate cyclase toxin (ACT) in the NPW of a representative wP-vaccinated baboon following challenge with pertussis is plotted against bacterial colonization (log_10_).

**Figure 4.**
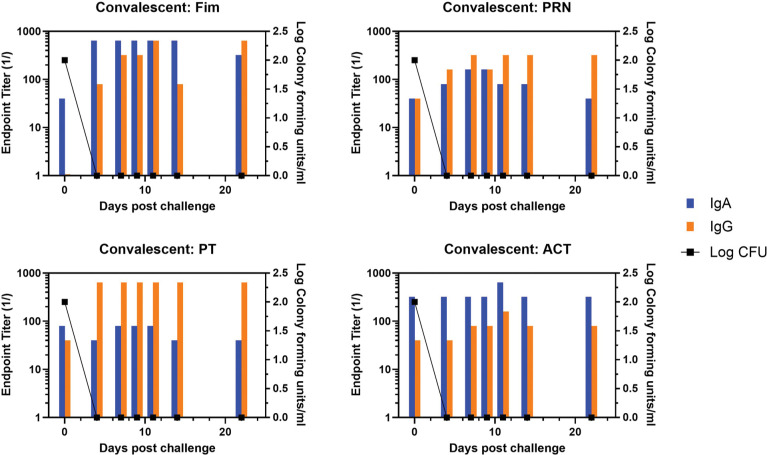
**Development of anti-pertussis antibody response and bacterial colonization in convalescent baboons following challenge**. Endpoint titers of IgA (blue bar) and IgG (orange bar) to fimbriae (Fim), pertactin (PRN), pertussis toxin (PT), and adenylate cyclase toxin (ACT) in the NPW of a representative convalescent baboon following challenge with pertussis is plotted against bacterial colonization (log_10_).

Representative animals are shown in Figure 2, with panels A and B representing each of the commercially available vaccines. A heat map of the responses of all animals is shown in Supplementary Figure 1. There was a trend for more IgA in response to one vaccine compared to the other, though the number of animals in each arm is too low to draw conclusions with significance only for PRN and Fim (*P*<0.02, *P*<0.03, respectively). As was noted in the original study, bacterial clearance was significantly delayed in the aP vaccine arm (>30 days). In contrast to aP-vaccinated animals, there was an accelerated IgA recall response in wP-vaccinated animals (*P*<0.007), except for PT (see Figure 3 for representative wP-vaccinated animal). Also, in contrast to aP vaccine recipients and naïve animals, wP-vaccinated animals showed accelerated clearance of bacteria (*P*<0.01). At the time of challenge, convalescent animals had a strong antibody response in the NPW to all antigens (representative in Figure 4). It should be noted that 4 of 6 challenged convalescent animals had no detectable colonization, whereas the other 2 animals (including the representative animal in Figure 4) had a very low bacterial load measured. A heat map of the response of all animals is shown in Supplementary Figure 1.

### The Effect of Antibody Response at the Time of Challenge on Bacterial Clearance

**Figure 5.**
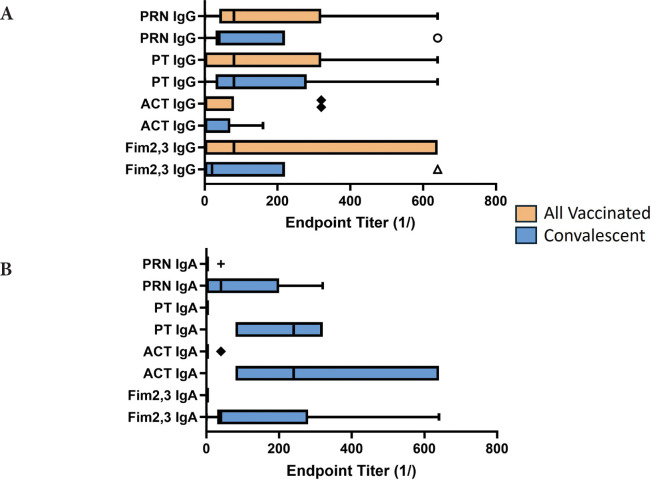
**Antibody response to pertussis antigens in NPW of vaccinated and convalescent animals**. Baboons were vaccinated with either aP or wP (orange bars) as described [9] or recovered from prior infection (convalescent, blue bars) and NPW collected just prior to challenge with *B. pertussis*. IgG (A) and IgA (B) antibody reactivity to pertussis antigens was measured by ELISA. Endpoint titers were determined and plotted as bar plots. Center lines show the medians; box limits indicate the 25th and 75th percentiles as determined by R software; whiskers extend 1.5 times the interquartile range from the 25th and 75th percentiles; outliers are represented by dots; crosses represent sample means; bars indicate 90% confidence intervals of the means. The differences between vaccine and convalescent animals reached significance for IgA responses to all antigens (Fim, *P*<0.049; ACT, *P*<0.001; PT, *P*<0.0002; PRN, *P*<0.029).

When measured at the time of challenge, similar to what has been published for the serum antibody response [9], all animals developed a mucosal IgG response to all vaccine antigens (Figure 5A). However, as shown in Figure 5B, only convalescent animals had a robust mucosal IgA response to pertussis antigens (*P* values range from <0.001-<0.06), and this correlated with protection from colonization. Within the vaccine recipients (aP and wP), there was no significant difference in antibody responses to vaccine antigens except for the lack of fimbrial response in animals receiving the 3-component vaccine. However, wP-vaccinated animals had an IgG response to ACT, as did convalescent animals, but not aP-vaccinated animals as that is not a component of the aP vaccine.

**Figure 6.**
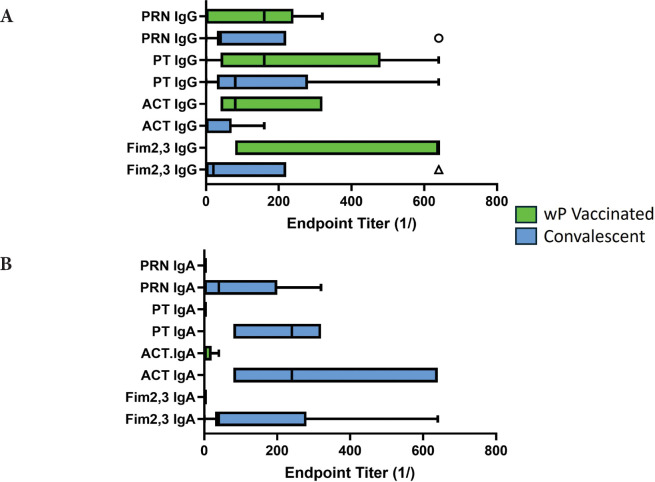
**Antibody response to pertussis antigens in NPW of wP-vaccinated and convalescent animals**. Baboons were vaccinated with wP (green bars) as described [9] or recovered from prior infection (convalescent, blue bars), and NPW collected just prior to challenge with *B. pertussis*. IgG (A) and IgA (B) antibody reactivity to pertussis antigens was measured by ELISA. Data was collected and reported as in Figure 1. The differences between wP and convalescent animals reached significance for IgA responses to ACT (*P*<0.028) and PT (*P*<0.003).

Given that both wP-vaccinated animals and convalescent animals reacted with antigens other than those in the aP vaccine, we compared the response of the wP-vaccinated animals and convalescent animals at the time of challenge; these results are shown in Figure 6. Convalescent animals had significantly lower IgG titers to ACT as compared to wP recipients (*P*<0.04) (Figure 6A). Strikingly, convalescent animals showed significantly higher IgA titers to ACT (*P*<0.01) and PT (*P*<0.001). The seemingly higher IgA titers for convalescent animals to Fim and PRN did not reach significance when compared to wP-vaccinated animals (Figure 6B). Convalescent animals with no colonies detected after challenge had higher IgA titers to ACT (*P*<0.04) and PT (*P*<0.007) at the time of challenge than those convalescent animals with bacteria positive NPW. In comparison, bacteria persisted for 12 to 21 days for wP-vaccinated (AUC 2.1x10^4^-1.1x10^7^) animals and 33 to 42 days in aP-vaccinated (AUC 3.9x10^6^-2.3x10^7^) animals.

## DISCUSSION

The whooping cough-like illness seen in baboons following experimental inoculation with *B. pertussis* resembles clinical disease in humans with features that include fever, leukocytosis, and the characteristic, chronic cough [8]. Similar to the protection from disease that is observed in humans following aP immunization, it has been shown that immunization with aP prevented clinical disease in vaccinated baboons upon challenge with *B. pertussis* [9]. However, aP vaccination results in colonization of the respiratory tract of aP-immunized baboons upon challenge. In contrast, unvaccinated baboons that recover from experimental pertussis infection are protected from disease with little to no colonization. We hypothesized that the mucosal antibody response in animals protected from disease differs from those with bacterial clearance. Analyses of the mucosal NPW antibody responses in vaccinated or convalescent baboons shown here demonstrate that mucosal IgA antibodies against pertussis antigens are key elements in the immune response seen in baboons with minimal bacteria colonization and should be considered as a goal for next-generation pertussis vaccines.

Both IgG and IgA are present in mucosal secretions. IgA is usually more effective on a molar basis at mucosal defense than IgG. The avidity of mucosal IgA, due to multimeric structure, enhances antibody binding with antigens and increases antibody mediated conformational or structural changes in the antigen. The diverse, elevated level of glycosylation of IgA antibodies, in comparison to IgG, further protects the mucosal surface by non-specific interference with microbial adherence. It should be noted that IgA in the NPW that were captured by pertussis antigens in the ELISA were equally detected by both anti-IgA and anti-J chain monoclonal antibodies (data not shown). This suggests that the dimeric IgA (dIgA) was produced locally, bound to the polymeric immunoglobulin receptor (pIgR) on the basolateral surface of epithelial cells, and transported through the cell to the apical or lumen side of the mucosa in the form of secretory IgA (sIgA).

There are limited studies on the human mucosal antibody response to *B. pertussis* during infection, convalescence, or in response to vaccination. Serum IgA from convalescent children inhibited adhesion of *B. pertussis* to ciliated epithelium; this was lacking in aP-vaccinated children [12]. Targeting *B. pertussis* to neutrophils using IgA or bispecific antibody enhanced clearance from the respiratory tract of mice [13]. IgA reactivity with sonicated bacteria was shown to increase as a function of time in humans infected with *B. pertussis* [14]. An early effort towards vaccination was application of a whole cell vaccine by aerosol into the nose of human participants [15]. There were fewer side effects, and IgA anti-pertussis titers were increased in respiratory secretions without evidence of a serum IgA response in comparison to intramuscular administration of the vaccine. Studies that are more recent have allowed determination of the effect of vaccine on not only disease but also colonization. A wP vaccine was engineered for reduced reactogenicity [16]. ot only was the vaccine less reactogenic, but it also induced antibody and cellular responses similar to standard wP vaccine. Mucosal administration, rather than systemic, of experimental vaccines, including outer membrane vesicles [17, 18] and live attenuated *B. pertussis* (BPZE1) [19] have been shown to reduce colonization in mice and which may involve mucosal IgA responses [20]. When evaluated in the baboon model, intranasal administration of the BPZE1 vaccine also resulted in significant reduction of *B. pertussis* colonization following challenge [21, 22]. Addition of novel adjuvants may also augment the ability of vaccines to suppress colonization [23] as well the use of virus-like particles [24]. Additional adjuvants or vaccines are needed to reprogram the immunity induced by the aP vaccine [25]. Together, the published results and those in this paper suggest that mucosal IgA responses are critical for preventing or reducing *B. pertussis* colonization. Conventional vaccines fail to induce mucosal IgA. Further studies on the contribution of individual pertussis antigens to this process are warranted.

Pertussis toxin is a major virulence factor that is functionally unique to *B. pertussis*. It is a hexameric A-B toxin consisting of 1 active subunit (S1) and 5 binding subunits (S2, S3, two S4, and S5). These binding subunits facilitate adhesion to extracellular glycoprotein receptors, including TLR4. Many clinical manifestations of pertussis, including but not limited to leukocytosis, are associated with the ADP-ribosylation activity of the S1 subunit. Pertussis toxoid (Ptx) is a critical component of all aP vaccines and has been used as a monocomponent vaccine. Most manufacturers of Ptx produce the toxoid by inactivation with formaldehyde; however, hydrogen peroxide is also used for inactivation, and it is less denaturing with enhanced preservation of tertiary and quaternary epitope structures as compared to formaldehyde [26]. Vaccine efficacy is higher with hydrogen peroxide-inactivated Ptx as compared to formaldehyde as either an aP component or monocomponent [27–30]. Incorporation of rPT into vaccines with or without other pertussis antigens was superior to standard aP vaccines at inducing PT neutralizing antibody responses including improved persistence (1 year) [31, 32]. Of interest, antibodies to specific potential protective PT epitopes may be preferentially elicited by natural infection compared to vaccine [33].

In addition to inhibiting immune function, ACT, in combination with PT, has a significant role in persistent colonization [34, 35]. In fact, it has been shown to contribute to the internalization of bacteria into nonphagocytic cells [36] by virtue of binding to CD11b/CD18 [37]. High titer antibody responses to ACT are found in sera from individuals that have been infected by *B. pertussis* or recipients of wP but not in aP recipients, as would be anticipated since ACT is not routinely found in aP vaccines [38–40]. Serum antibodies with ACT neutralization activity were also seen after infection of humans and baboons [41]. ACT neutralizing antibodies promote phagocytosis and confer protection *in vivo* [42, 43]. It has been shown that the C-terminal RTX domain is immunodominant, elicits neutralizing antibody [44, 45], and may enhance vaccine protection [46]. Mapping studies should identify potential protective epitopes [47, 48].

Fimbrial antigens belong to the type I pili family, are expressed on the bacterial surface, contribute to adhesion of the bacteria to the ciliated epithelium of the respiratory tract, and cooperate with FHA to suppress inflammation in response to infection [49, 50]. There are 2 serologically distinct Fim antigens: Fim2 and Fim3. Five component aP vaccines (and wP vaccine), but not 3 component aP, include Fim2/Fim3. Antibodies to Fim are elicited by natural infection or immunization with wP and 5 component aP vaccines. Fim-specific sera antibodies and monoclonal anti-Fim antibodies have been shown to reduce *B. pertussis* adherence to epithelia [51, 52]. It has also been shown that increasing the Fim2/Fim3 content of aP vaccines improves the protective efficacy of the vaccine [53]. Together, these results suggest that a mucosal antibody response to Fim should contribute to preventing colonization, and Fim2/Fim3 should be included in vaccines [54].

Evidence has also accumulated supporting a role for specific T cell-mediated responses and association with disease prevention and bacterial clearance in humans [55] and baboon models [8]. Response to infection and vaccination with wP vaccine is skewed towards Th1/Th17, while the response to aP is mostly Th2. This may suggest that a Th2 response may be sufficient for protection from disease, but a Th1 response is required for bacterial clearance. IL-17-producing tissue resident memory T cells (T_RM_) may play a key role in long-term memory. Protective T_RM_ are induced during natural infection and by immunization with wP vaccine but not aP vaccines [56], which may explain waning immunity following aP vaccination. These T_RM_ have been shown to persist in adults vaccinated with wP vaccine as children [57]. Of importance here, IL-17-producing Th17 cells support class switching to IgA and upregulating the polymeric immunoglobulin receptor, and elevated levels of IgA have been shown when Th17 are prominent [58]. Mucosal immunization with test vaccine/antigens and adjuvant induced both Th17 cells and IgA antibody and protected against nasal colonization in murine models [20]. Discerning the protective antibody or antigens in the mucosal immune response to *B. pertussis* will contribute to more effective vaccine strategies. However, in addition to identifying antigen targets, more advances in mucosal adjuvants and strategies are needed [59, 60].

## SUPPLEMENTARY DATA

Supplementary materials are available at the *Pathogens and Immunity* website. Supplementary data may be provided by the authors to benefit the reader. Supplementary data are not copyedited and are the sole responsibility of the authors. Questions or comments related to supplementary materials should be addressed to the corresponding author.
