# Supplementary material for: Mucosal IgA Antibodies are Critical for Bacterial Clearance of Bordetella pertussis in the Baboon Model
Source: Pathog Immun. 2025 Jun 13;10(2):126–45. doi: 10.20411/pai.v10i2.800 (PMC12225615; doi:10.20411/pai.v10i2.800)
Supplement: Supplementary Figures [file pai-10-126-s01.pdf]

PATHOGENS AND IMMUNITY
IN ASSOCIATION WITH CASE WESTERN RESERVE UNIVERSITY AND THE RICHARD J. FASENMYER FOUNDATION

Supplementary Figure 1A

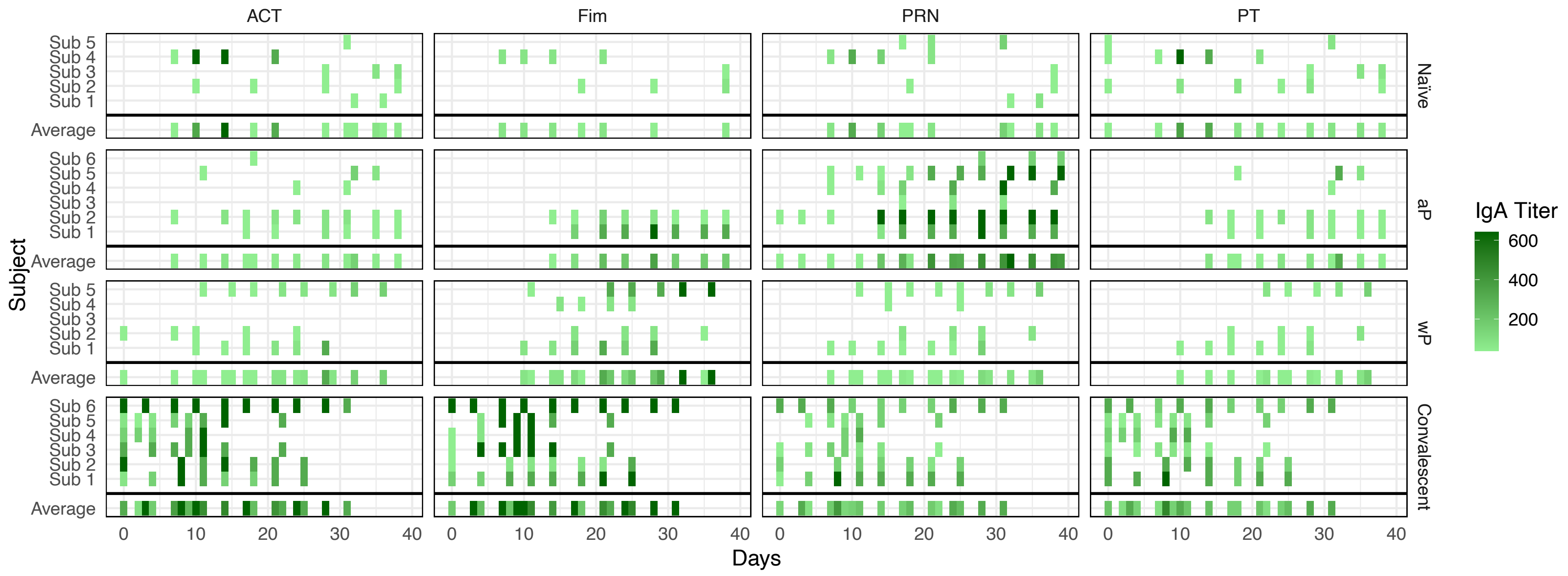

Supplementary Figure 1A-C. Development of the anti-pertussis antibody response and bacterial colonization in baboons following challenge. Heatmap of endpoint titers of IgA **(A)** and IgG **(B)** against fimbriae (Fim), pertactin (PRN), pertussis toxin (PT), and adenylate cyclase toxin (ACT) across days post-challenge in animal groups receiving either the aP (n=6) or wP (=5) vaccine, convalescent (n=6), or naïve (n=5). Bacterial conoloization **(C)** (days post-challenge) for each participant in the study.

Chauhan G, Gawron MA, Belli AJ, Reimann KA, Schneider R, Wang Y, Klempner MS, Cavacini LA. Mucosal IgA Antibodies are Critical for Bacterial Clearance of *Bordetella pertussis* in the Baboon Model. *Pathogens and Immunity*. 2025;10(2):126–145. doi: 10.20411/pai.v10i2.800

PATHOGENS AND IMMUNITY
In Association with Case Western Reserve University and The Richard J. Fasenmyer Foundation

Supplementary Figure 1B

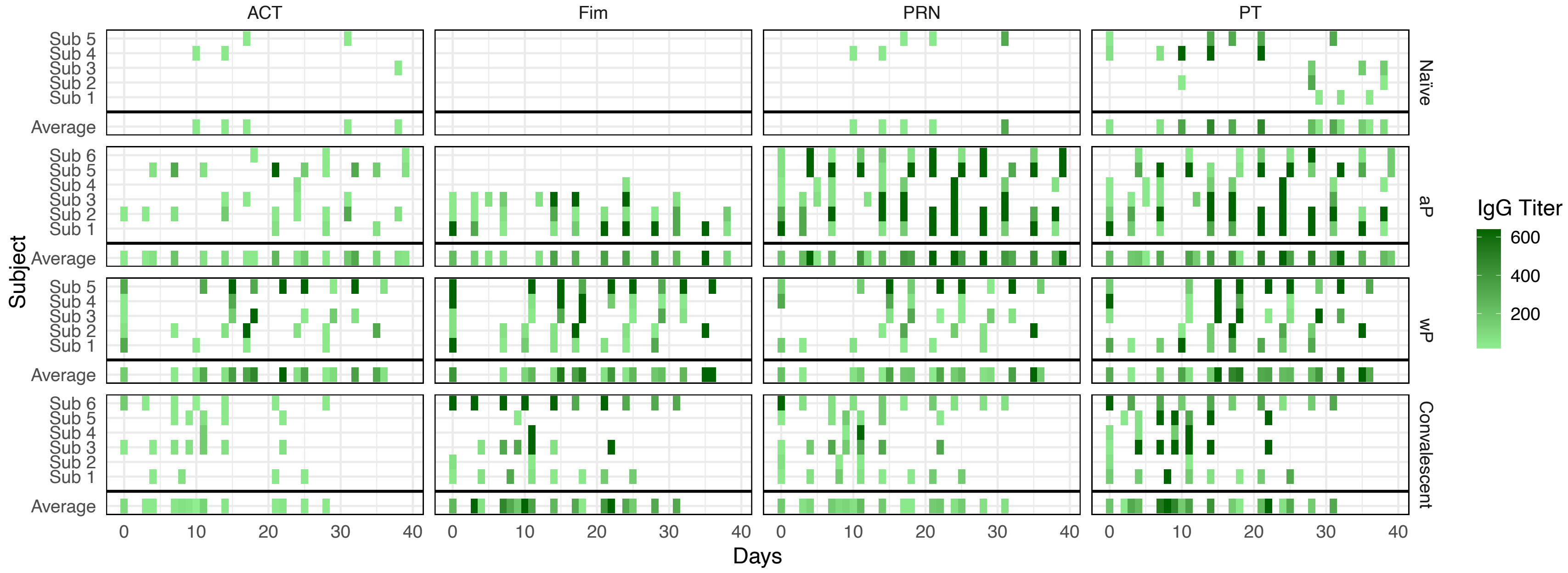

Chauhan G, Gawron MA, Belli AJ, Reimann KA, Schneider R, Wang Y, Klempner MS, Cavacini LA. Mucosal IgA Antibodies are Critical for Bacterial Clearance of *Bordetella pertussis* in the Baboon Model. *Pathogens and Immunity*. 2025;10(2):126–145. doi: 10.20411/pai.v10i2.800

PATHOGENS AND IMMUNITY
IN ASSOCIATION WITH CASE WESTERN RESERVE UNIVERSITY AND THE RICHARD J. FASENMYER FOUNDATION

Supplementary Figure 1C

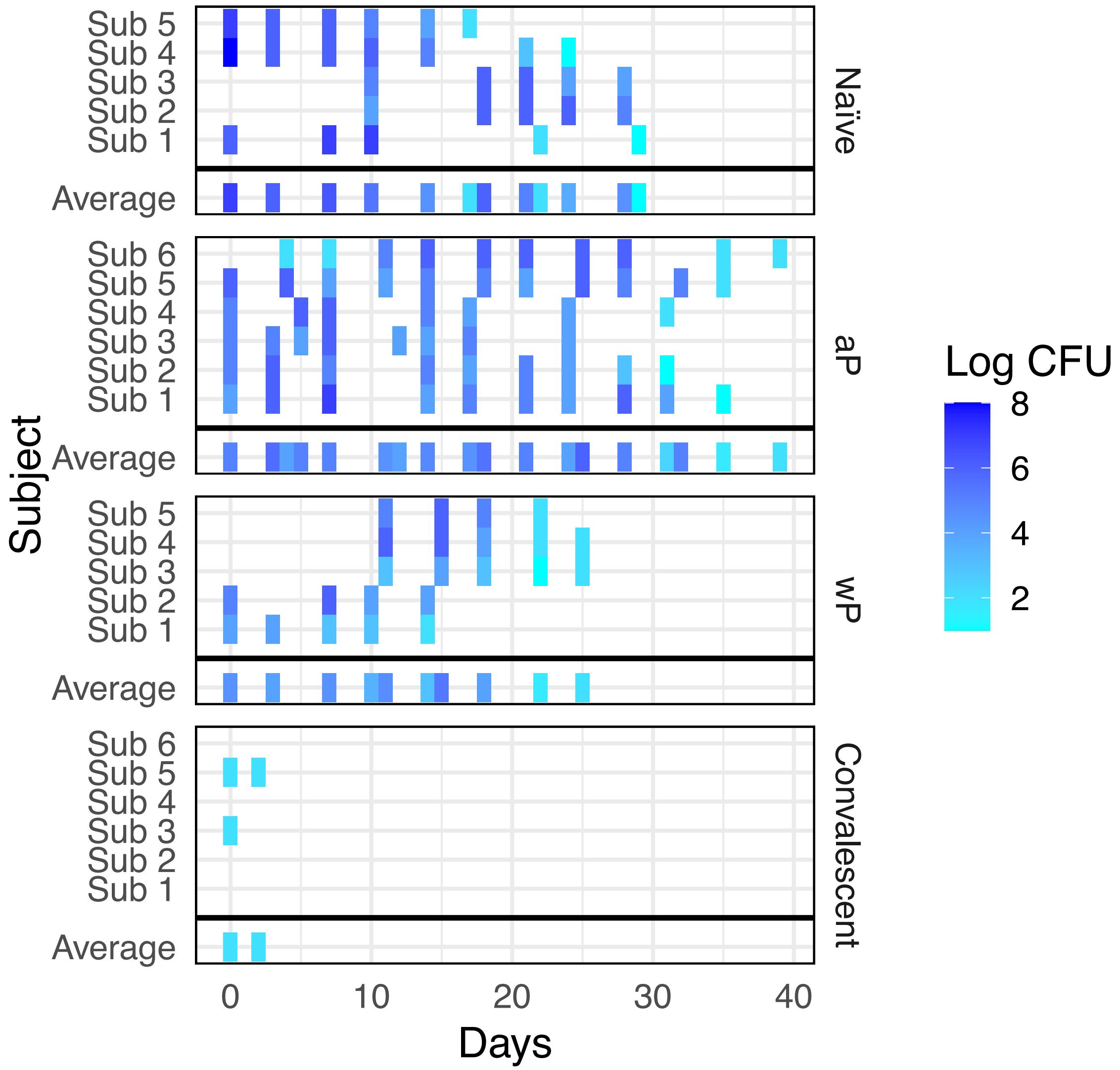

Chauhan G, Gawron MA, Belli AJ, Reimann KA, Schneider R, Wang Y, Klempner MS, Cavacini LA. Mucosal IgA Antibodies are Critical for Bacterial Clearance of *Bordetella pertussis* in the Baboon Model. *Pathogens and Immunity*. 2025;10(2):126–145. doi: 10.20411/pai.v10i2.800
